# Construction and validation of a folate metabolism-related gene signature for predicting prognosis in HNSCC

**DOI:** 10.1007/s00432-024-05731-4

**Published:** 2024-04-16

**Authors:** Lu Wang, Ye He, Yijiang Bai, Shuai Zhang, Bo Pang, Anhai Chen, Xuewen Wu

**Affiliations:** Department of Otolaryngology Head and Neck Surgery, Xiangya Hospital of Central South University, 87 Xiangya Road, Changsha, 410008 Hunan China

**Keywords:** Head and neck squamous cell carcinoma (HNSCC), Serine hydroxymethyltransferase 2 (SHMT2), Folate one-carbon metabolism, Tumor microenvironment

## Abstract

**Purpose:**

Metabolic reprogramming is currently considered a hallmark of tumor and immune development. It is obviously of interest to identify metabolic enzymes that are associated with clinical prognosis in head and neck squamous cell carcinomas (HNSCC).

**Methods:**

Candidate genes were screened to construct folate metabolism scores by Cox regression analysis. Functional enrichment between high- and low-folate metabolism groups was explored by GO, KEGG, GSVA, and ssGSEA. EPIC, MCPcounter, and xCell were utilized to explore immune cell infiltration between high- and low-folate metabolism groups. Relevant metabolic scores were calculated and visually analyzed by the “IOBR” software package.

**Results:**

To investigate the mechanism behind metabolic reprogramming of HNSCC, 2886 human genes associated with 86 metabolic pathways were selected. Folate metabolism is significantly enriched in HNSCC, and that the six-gene (MTHFD1L, MTHFD2, SHMT2, ATIC, MTFMT, and MTHFS) folate score accurately predicts and differentiates folate metabolism levels. Reprogramming of folate metabolism affects CD8T cell infiltration and induces immune escape through the MIF signaling pathway. Further research found that SHMT2, an enzyme involved in folate metabolism, inhibits CD8T cell infiltration and induces immune escape by regulating the MIF/CD44 signaling axis, which in turn promotes HNSCC progression.

**Conclusions:**

Our study identified a novel and robust folate metabolic signature. A folate metabolic signature comprising six genes was effective in assessing the prognosis and reflecting the immune status of HNSCC patients. The target molecule of folate metabolic reprogramming, SHMT2, probably plays a very important role in HNSCC development and immune escape.

**Supplementary Information:**

The online version contains supplementary material available at 10.1007/s00432-024-05731-4.

## Introduction

Head and neck squamous cell carcinoma (HNSCC) originating from mucosal epithelial cells of the larynx, pharynx, and oral cavity is the sixth most common malignancy worldwide, with approximately 600,000 cases and 380,000 deaths annually (Ferlay et al. 2015; Johnson et al. 2020). Most patients are diagnosed in the late stage of HNSCC, and the prognosis is usually poor. Despite good progress in screening, diagnosis and multimodal treatment, the 5-year overall survival rate is still less than 50% (Cohen et al. 2016). Metabolic reprogramming is currently considered one of the important markers of tumor immune escape. However, the specific mechanism by which tumor growth and proliferation rely on tumor metabolic pathways to promote immune escape is not yet clear.

The folate cycle, also known as one-carbon metabolism, primarily supports cellular nucleotide supply, S-adenosylmethionine production, and amino acid homeostasis (Ducker and Rabinowitz 2017). High folate levels are usually positively associated with markers of genomic stability and a lower risk of colorectal cancer, but in trials, folate interventions did not reduce the risk of cancer (Kim 2007). There are results showing that artificial folic acid supplementation further promotes cancer cell growth in animal models, human trials, and cancer incidence data (Cole et al. 2007). Several large-scale human trials have shown that folic acid supplementation increases the risk of breast, colon, lung and prostate cancers (Cole et al. 2007; Ebbing et al. 2009; Figueiredo et al. 2009; Stolzenberg-Solomon et al. 2006). The role that folic acid plays in HNSCC and the specific mechanisms involved need to be further clarified. Serine hydroxymethyltransferase 2 (SHMT2) is localized in mitochondria, the rate-limiting enzyme of the one-carbon unit (Tibbetts and Appling 2010). Tumor cells can alter these metabolic enzymes to maintain the supply of one-carbon units needed for proliferation. Recent studies have shown that overexpression of SHMT2 is observed in a variety of cancers, including breast, melanoma, lung, ovarian, and prostate cancers (Anderson et al. 2011; Ding et al. 2013; Lee et al. 2014), and that it is associated with tumorigenesis and progression. SHMT2 has been shown in clinical studies that the higher the expression, the more aggressive the tumor is and the worse the prognosis (Jin et al. 2022). Therefore, SHMT2 is an important potential target for metabolic reprogramming in tumors and its role in tumors needs to be further explored.

We found that folate underwent metabolic reprogramming in HNSCC by analyzing bulk transcriptome data and explored the relationship between folate metabolic reprogramming and tumor immunity. We further found that folate metabolic reprogramming affected CD8T cell infiltration through the MIF signaling pathway by analyzing single-cell data. More importantly, we found that the target molecule of folate metabolic reprogramming, SHMT2, plays a key role in HNSCC development and immune escape. Our study fills the gap of SHMT2 in HNSCC.

## Methods

### Metabolic transcript GSVA and construction of folate score

We selected 2886 metabolism-related genes from the Kyoto Encyclopedia of Genes and Genomes (KEGG) database and estimated the enrichment scores of metabolic pathways for each sample in the TCGA cohort based on GSVA. Differential analysis of 20 folate metabolism-related genes (the one-carbon pool by folate and the folate biosynthesis pathway) between cancer and adjacent tissues revealed that 16 genes were differentially expressed in HNSCC (adjusted P-values < 0.05 and log fold change > 1). Subsequently, we employed univariate regression and stepwise Cox regression analysis on 16 folate metabolism-associated genes and a folate score was constructed. Schoenfeld residuals were used to test the proportional hazards assumption of Cox regression models. We obtained a validation dataset, GSE65858, with clinical annotations and overall survival (OS) information from the GEO database in the latest manuscript. The expression profile includes 270 HNSCC samples. Patients were categorized into a low-folate group and a high-folate group, with the median folate as the cutoff point. Kaplan–Meier plots of OS and DFS were generated using R package survival. The log-rank test *p* < 0.05 was used to determine differences in survival times.

### Functional analysis

GO and KEGG analyses were performed using the “clusterProfiler” R package to enrich DEG into relevant pathways. In addition, the activation level of 50 HALLMARK pathways was estimated by the ssGSEA R package. Various metabolic scores were calculated based on the work of Smiraglia et al. (Parsa et al. 2020).

### Immune cell infiltration assessment

TIMER, EPIC, MCPcounter, CIBERSORT, QUANTISEQ, and xCell algorithms were used to estimate the degree of immune cell infiltration. The above algorithm is included in the “IOBR” R package.

### Single-cell data processing

We re-annotated the cell types of GEO: GSE151530. Specifically, malignant, fibroblasts, CD8T, CD4T, endothelial, mono, plasma, mast, and myocyte can be identified by uniform manifold approximation and projection (UMAP). Cell–cell ligand receptor analysis was performed using the CellChat package. Pseduobulk RNA-seq analysis was performed by subsetting the raw counts for each sample.

### Cell lines

Normal human oral mucosa precancerous lesion cell line (DOK) and several human HNSCC cell lines (SCC-4, HN8, and FaDu) were obtained from otolaryngology department of Xiangya Hospital. DOK and HN8 cells were cultured in RPMI-1640 medium containing 10% fetal bovine serum (FBS). The culture medium for the SCC-4 and FaDu was Dulbecco’s modified Eagle medium (DMEM) with 10% FBS. All cells were cultured at 37 °C with 5% CO_2_.

### Immunohistochemistry

Formalin solution (10%) fixed surgically obtained head and neck squamous cell carcinoma, embedded in paraffin, and sectioned at a thickness of 3 to 5 μm. The slides were washed with 3% hydrogen peroxide (H2O2) to block endogenous peroxidase activity and to avoid immunoreaction. The tissue was blocked in 5% BSA, and the slides were incubated with primary antibodies (SHMT2, ProteinTech, 11099-1-AP, 1:100) overnight at 4 °C. After washing with PBS, the sections were incubated with appropriate reaction enhancement solution and enhanced HRP-conjugated sheep anti-mouse/rabbit IgG polymer (ZSGB-Bio) and 3,3’-diaminobenzidine (DAB; ZSGB-Bio) substrate solution to visualize the immunoreaction. The nuclei were stained with hematoxylin (ZSGB-Bio) and the differentiation solution was decolorized and observed under a microscope.

### Western blotting analysis

The cell protein samples were extracted using RIPA lysis buffer containing protease inhibitors (K1007, APE × BIO), and the protein concentration was determined by a bicinchoninic acid assay kit (20201ES76, YEASEN). The quantified proteins were electrophoresed using 10% SDS–polyacrylamide gel and transferred to PVDF membrane. The PVDF membrane was blocked with 5% skim milk for 1 h at room temperature, and then incubated with the primary antibody at 4 °C overnight. The primary antibody was antibody against SHMT2 (proteintech Cat#11099-1-AP, 1:3000) and antibody against GAPDH (Proteintech Cat# 60004-1-Ig, 1:1000). After incubation with horseradish peroxidase-conjugated anti-mouse or anti-rabbit secondary antibodies, band images were digitally captured and quantified using enhanced chemiluminescence.

### Statistical analysis

The results were expressed as mean ± standard error of the mean. The *t*-test was used to compare the differences between the two groups, and one-way ANOVA was used to compare differences between multiple groups. In addition, we used the Benjamini–Hochberg false discovery rate (FDR) approach (*p* < 0.05) to control for multiple comparisons between the groups. The level of significance was *p* < 0.05 (*p < 0.05, **p < 0.01, and ***p < 0.001).

## Results

### Bulk transcriptomic profiling revealed evident folate metabolism dysregulation in HNSCC

**Fig. 1:**
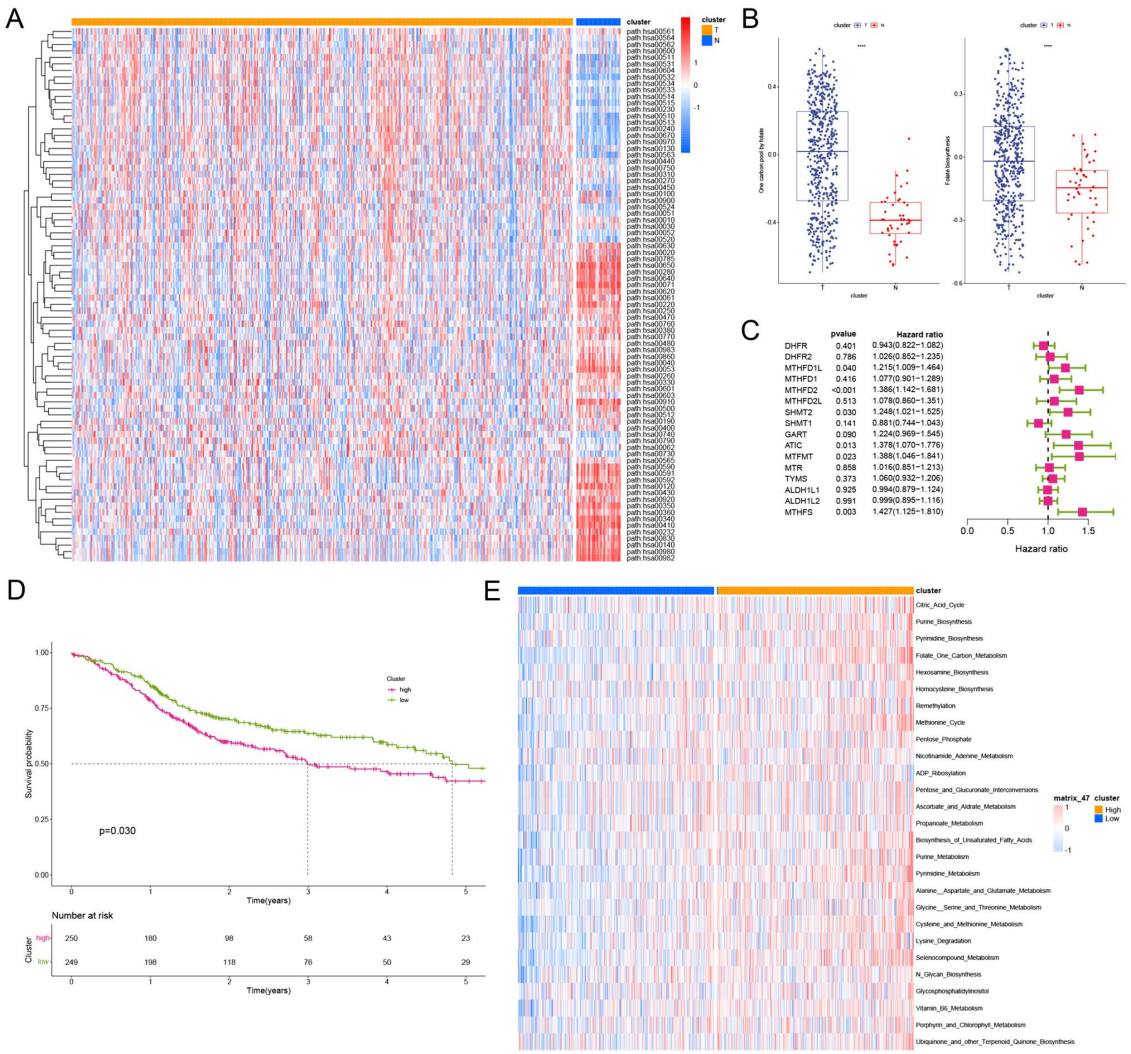
Bulk transcriptomic profiling revealed evident folate metabolism dysregulation in HNSC. **A** KEGG metabolic pathway enrichment heat map. **B** Box plots presenting significant differences in folate metabolism. **C** Folate metabolism-related genes were analyzed by one-way COX regression. **D** K–M curves for folate metabolism scores. **E** Top 20 pathways with high- and low-folate metabolism score difference in REACTOME database

To explore the mechanism behind the metabolic reprogramming of HNSCC to promote tumor progression, we selected 2886 metabolism-related genes from the Kyoto Encyclopedia of Genes and Genomes (KEGG) database. To reveal the metabolic heterogeneity of HNSCC, we estimated the enrichment scores of metabolic pathways for each sample in the TCGA cohort based on GSVA (Fig. 1A; Supplementary Table 1). One carbon pool by folate and the folate biosynthesis pathway were significantly enriched in HNSCC (Fig. 1B). Differential analysis of 20 folate metabolism-related genes (the one-carbon pool by folate and the Folate biosynthesis pathway) between cancer and adjacent tissues revealed that 16 genes were differentially expressed in HNSC. Subsequently, we analyzed the prognostic significance of these 16 genes through univariate Cox regression (Fig. 1C). The results suggested that high expression of MTHFD1L, MTHFD2, SHMT2, ATIC, MTFMT, and MTHFS predicted a poor prognosis. Therefore, we constructed and validated a folate score based on the above six genes through stepwise multiple regression analysis, and the higher the folate score is, the worse the prognosis of the patient (Supplementary Fig. 1 and Fig. 1D). The folate metabolism signature we constructed also has good predictive ability in the validation cohort (Supplementary Fig. 2). More importantly, we analyzed the relationship between the folate score and the known associated metabolic signatures and found that folate-related metabolic pathways (folate one-carbon metabolism, purine metabolism, and pyrimidine metabolism) were significantly enriched in the high-folate score group (Fig. 1E). Taken together, our results suggest that folate metabolism is significantly enriched in HNSCC, and that the six-gene folate score we constructed accurately predicts and differentiates folate metabolism levels.

### The relationship between folate metabolism reprogramming and immunity in HNSCC

**Fig. 2:**
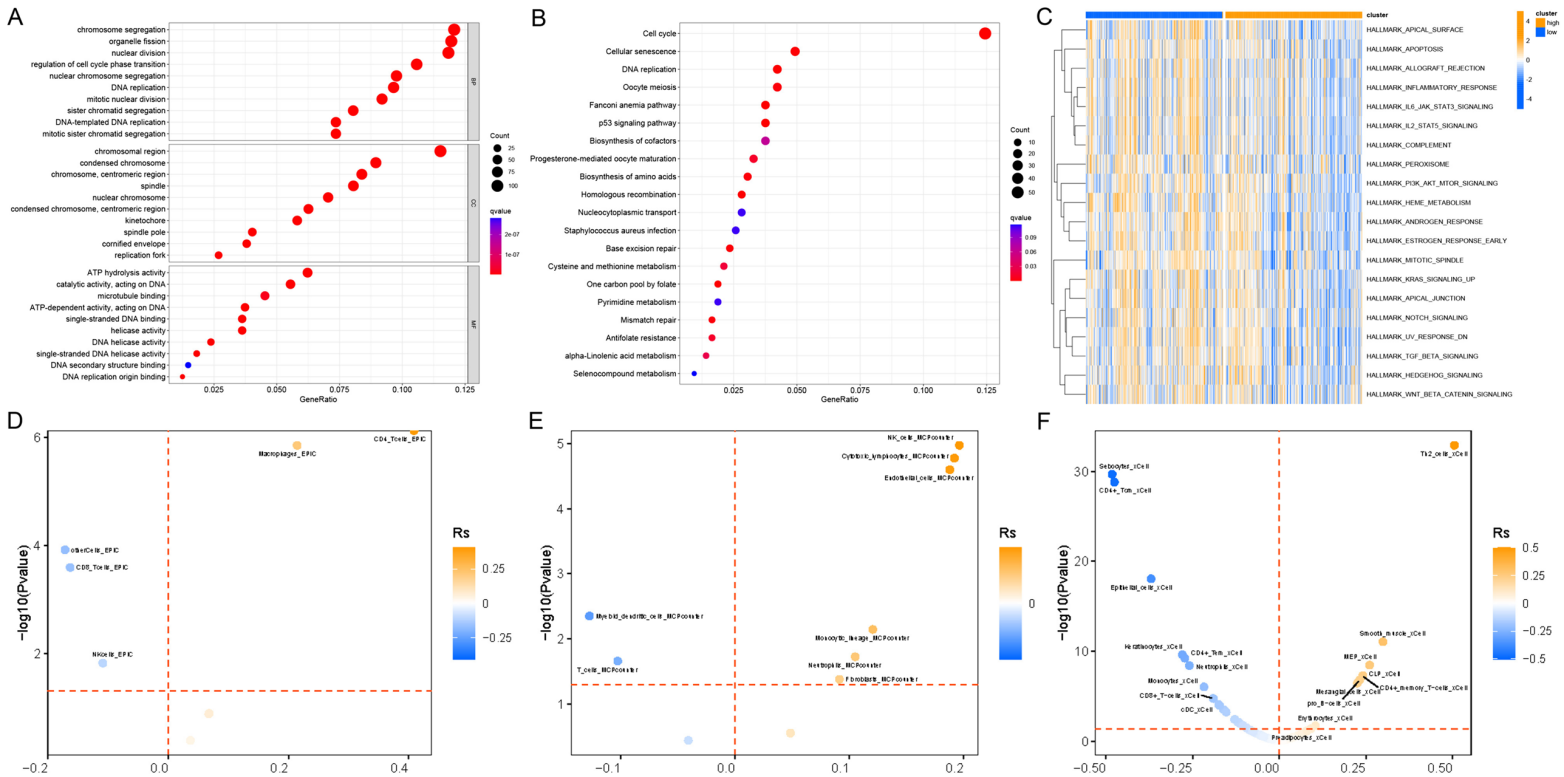
The relationship between folate metabolism reprogramming and immunity in HNSCC. **A** GO was used to analyze the enrichment between high- and low-folate metabolism groups. **B** KEGG was used to analyze the enrichment between high- and low-folate metabolism groups. **C** Analysis of hallmark enrichment between high- and low-folate metabolism groups. Box plots presenting significant differences in EPIC (**D**), MCPcounter (**E**), and xCell (**F**)

To further explore the specific mechanisms by which reprogramming of folate metabolism in HNSCC affects prognosis, we analyzed the folate score by enriching for differential genes. The GO (Fig. 2A) and KEGG (Fig. 2B) analysis results showed that folate metabolism was significantly associated with DNA damage and repair. In addition, the folate-related pathways were significantly enriched in KEGG results, further validating the accuracy of our constructed folate score. More importantly, the Hallmark enrichment analysis showed that folate metabolism was significantly correlated with immune-related pathways, and immune-related pathway scores were significantly under-expressed in the high-folate score group, suggesting suppression (Fig. 2C). To further explore the potential mechanisms of immune suppression by reprogramming of folate metabolism, we analyzed by different immune cell infiltration algorithms (EPIC, MCPcounter, and xCell) and found that folate scores were significantly negatively correlated with CD8T cell infiltration, with higher folate scores being associated with lower CD8T cell infiltration scores (Fig. 2D–F).

### Single-cell data reveal reprogramming of folate metabolism affects CD8T cell infiltration through the MIF signaling pathway

**Fig. 3:**
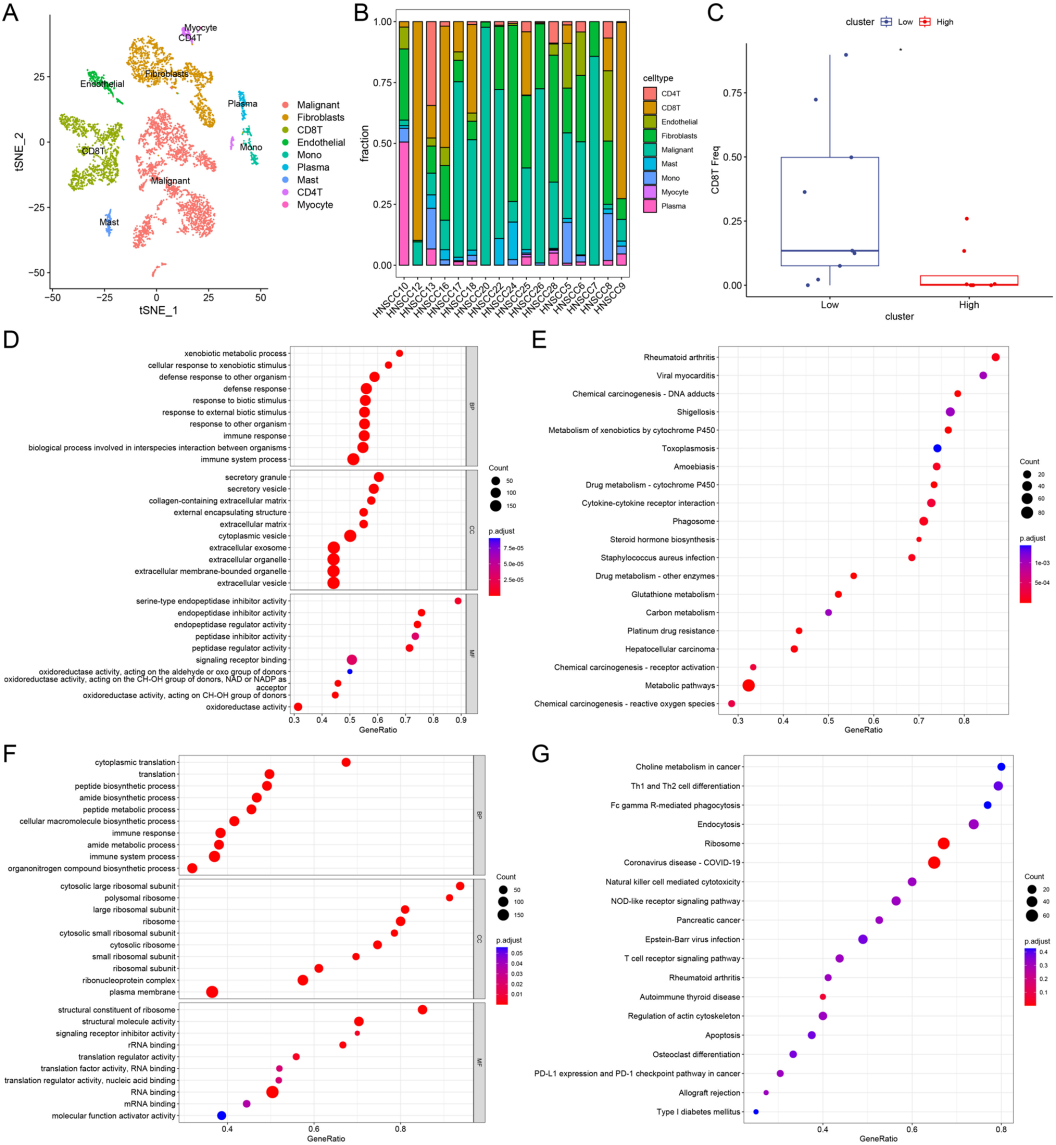
Single-cell data revealed reprogramming of folate metabolism affects CD8T cell infiltration. A-B, Relabeling of GSE103322 single-cell data (**A**) to analyze the proportion of each cell type in different patients (**B**). **C** By converting single-cell data into bulk data, significant differences in CD8T cell infiltration revealed by pesudobulk differential expression analysis. GO (**D**) and KEGG (**E**) were used to analyze the enrichment between high- and low-folate metabolism groups in cancer cells. GO (**F**) and KEGG (**G**) were used to analyze the enrichment between high- and low-folate metabolism groups in CD8T cell

**Fig. 4:**
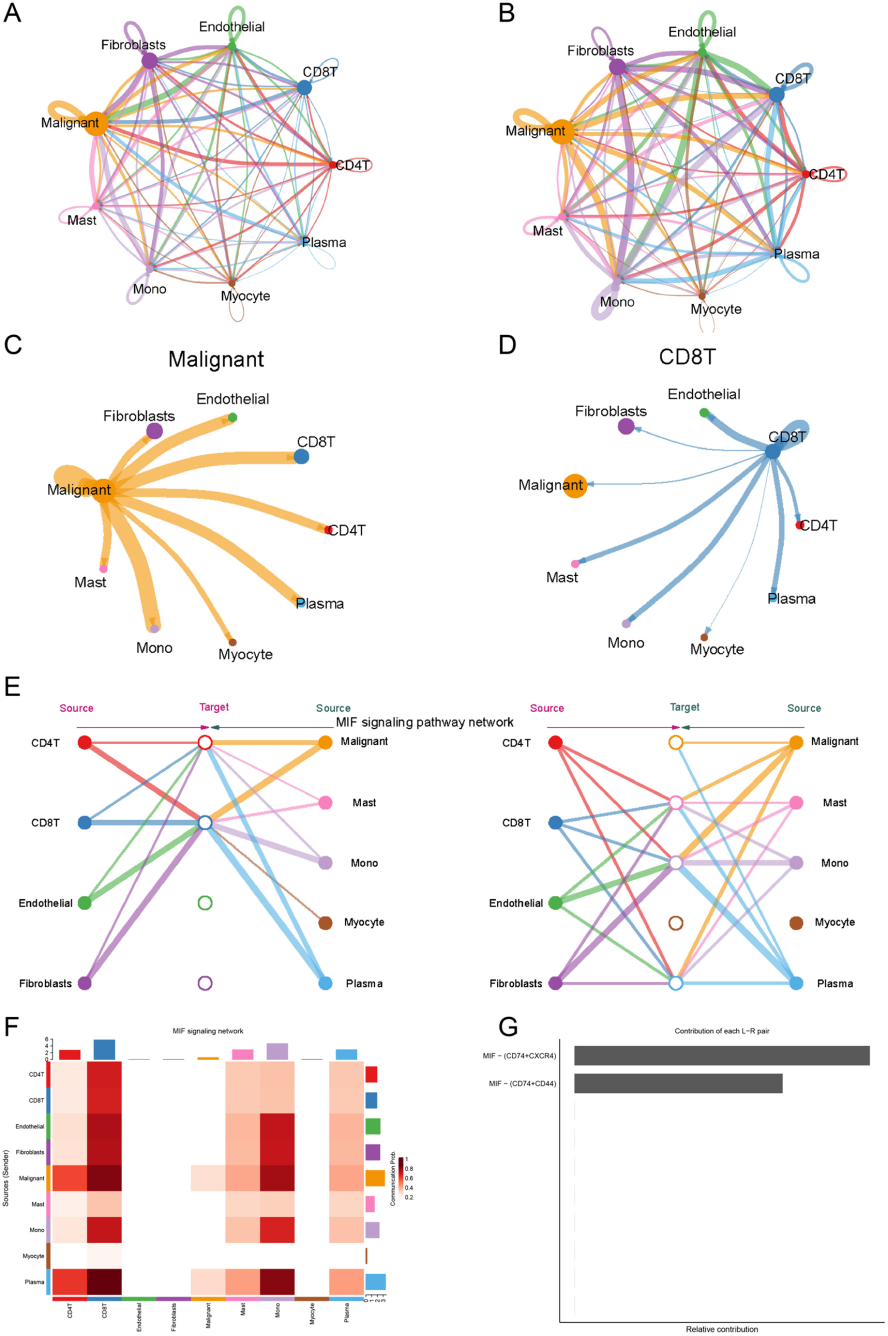
Single-cell data revealed cancer cell affects CD8T cell infiltration through the MIF signaling pathway. **A**, **B**, Analyzing cellular communication through CellChat. **C**, **D** show the cellular communication between tumor cells and CD8T cells. **E**, **F** Communication target analysis revealed a significant enrichment of the MIF signaling pathway. **G** The main targets of the MIF signaling pathway were analyzed

To further clarify the specific mechanisms by which reprogramming of folate metabolism regulates immune escape, we re-annotated the GSE103322 single-cell data and analyzed the proportion of each type of cells among different patients (Fig. 3A, B). We found that CD8T cell infiltration was significantly reduced in patients with high-folate scores by pesudobulk differential expression analysis (Fig. 3C). Further enrichment analysis revealed that reprogramming of folate metabolism was significantly associated with immune responses in HNSCC tumor cells (Fig. 3D, E). More importantly, in CD8T cells, folate metabolic reprogramming was significantly associated with T cell receptors and immune responses (Fig. 3F, G). Next, we analyzed the cellular communication by CellChat, and the highest percentage of secreted signaling was found in HNSCC (Fig. 4A, B). There was strong cellular communication between tumor cells and CD8T cells, and it was mainly the tumor cells that sent signals, not the CD8T cells (Fig. 4C, D). Communication target analysis revealed a significant enrichment of the MIF signaling pathway, and our results suggested that tumor cells acted as senders of the MIF signaling pathway, while CD8T cells acted as receivers of the signals (Fig. 4E, F; Supplementary Table 2). We also analyzed the principal targets of the MIF signaling pathway and found significant enrichment of MIF-CD74/CXCR4/CD44 (Fig. 4G). In summary, our results suggest that reprogramming of folate metabolism affects CD8T cell infiltration and induces immune escape through the MIF signaling pathway.

### The role of SHMT2, a target molecule for regulating folate metabolism reprogramming, in the occurrence and development of HNSCC

**Fig. 5:**
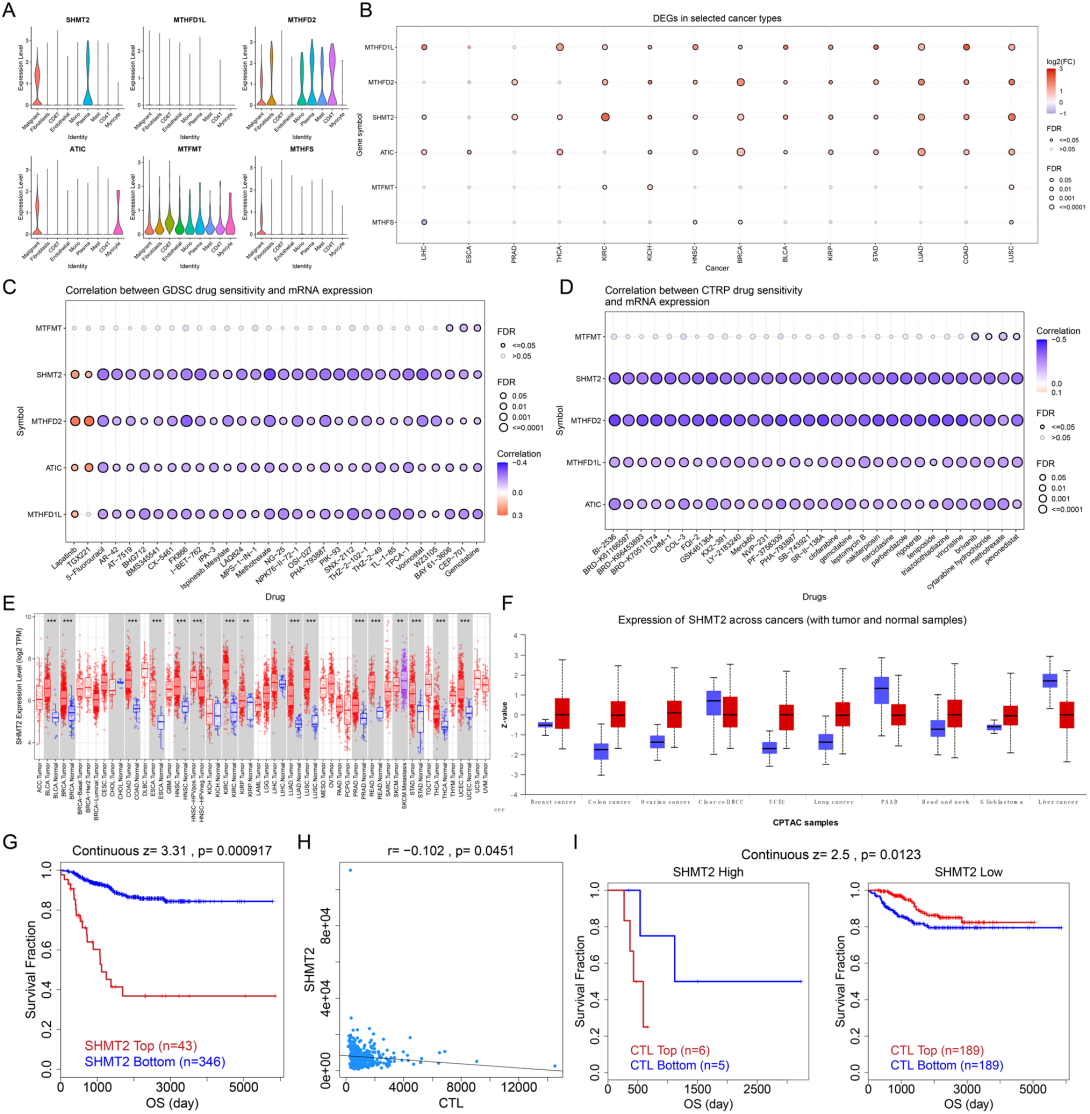
The role of SHMT2, a target molecule for regulating folate metabolism reprogramming, in the occurrence and development of HNSC. **A** The violin plot shows the expression of target genes in single-cell data. **B** The bubble chart displays the differential expression of target genes in pan-cancer data. **C**, **D** The bubble chart shows the correlation between target genes and drug sensitivity. **E**, **F**, Box plots show the expression of SHMT2 at the transcriptional and protein levels in pan-cancer data. **G** K–M curves for SHMT2. **H** The scatter plot shows the correlation between SHMT2 and CTL. **I** K–M curves for SHMT2 and CTL

To clarify the target molecules that regulate reprogramming of folate metabolism, we analyzed the expression of six folate metabolism-related genes in individual cells. The results of single-cell data suggested that SHMT2, ATIC and MTHFS were mainly expressed in tumor cells (Fig. 5A). Further analysis revealed that SHMT2 was highly expressed in several tumors (Fig. 5B), and drug sensitivity analyses of both GDSC (Fig. 5C) and CTRP (Fig. 5D) databases were performed, and the RNA expression level of SHMT2 was significantly negatively correlated with most drug sensitivities. The transcriptome (Fig. 5E) and proteome (Fig. 5F) of SHMT2 pan-cancer were further validated, and SHMT2 was found to be generally highly expressed in tumors. By analyzing the E-MTAB-179 data, it was found that high SHMT2 expression suggested poor prognosis and was negatively correlated with CTL (Fig. 5G, H). What is more, CTL infiltration in the SHMT2 low-expression group suggested a good prognosis (Fig. 5I), while CTL infiltration in the SHMT2 high-expression group suggested a poor prognosis, suggesting that SHMT2 was associated with T cell depletion.

### The role of the MIF signal axis in the occurrence and development of HNSCC

**Fig. 6:**
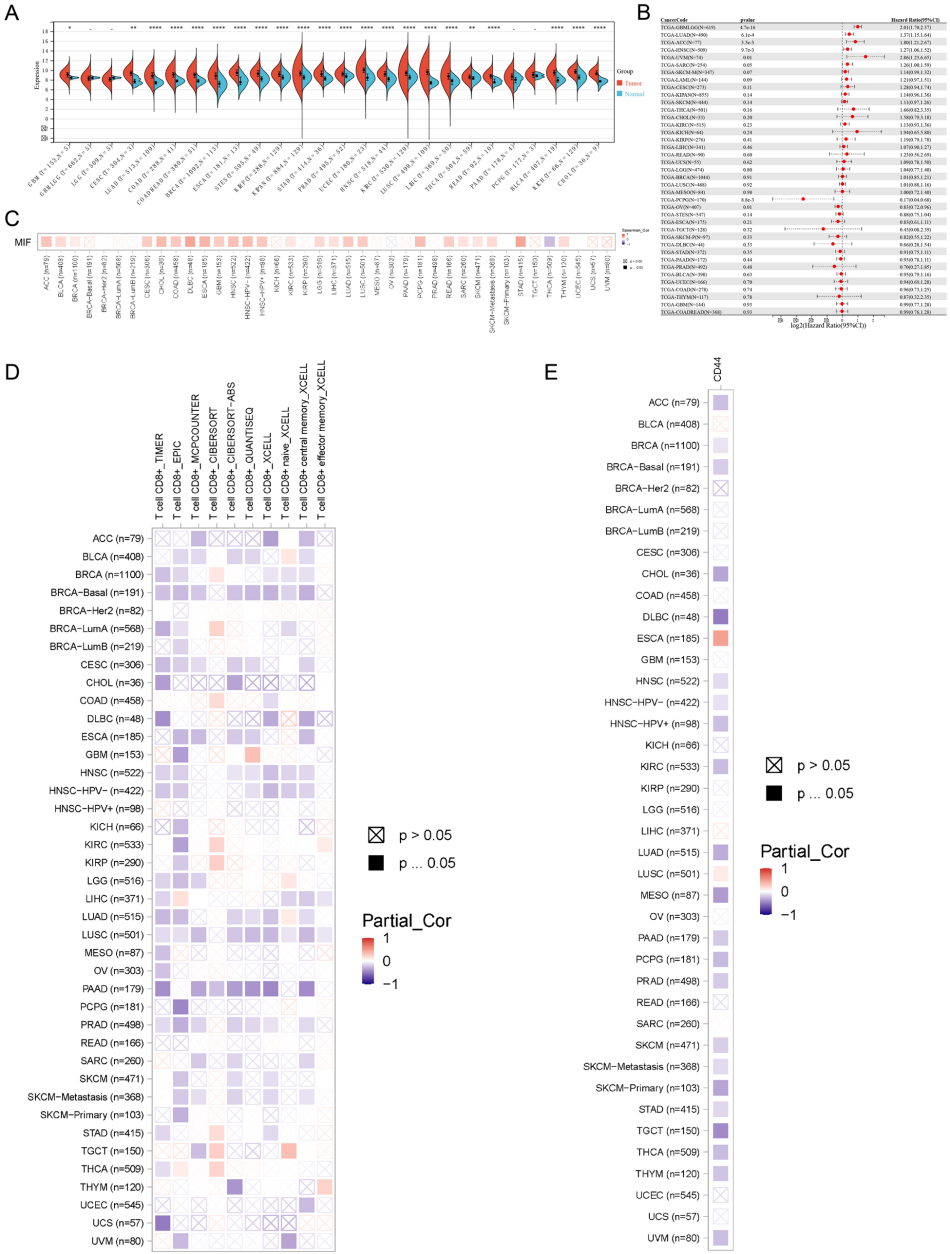
The role of the MIF signal axis in the occurrence and development of HNSCC. **A** Box plots show the expression of MIF at the transcriptional levels in pan-cancer data. **B** The forest map presents the prognosis of MIF in pan-cancer data. **C** The heatmap shows the correlation between MIF and SHMT2. **D** The heatmap shows the correlation between MIF and CD8T cell. **E** The heatmap shows the correlation between MIF and CD44

We also analyzed the expression of the communication target MIF in individual tumors (Fig. 6A), and MIF was significantly overexpressed in several tumors including HNSCC. The high expression of MIF was suggestive of poor prognosis in several tumors including HNSCC (Fig. 6B). In addition, SHMT2 was significantly positively correlated with MIF in the vast majority of tumors (Fig. 6C). We also analyzed the relationship between SHMT2 and CD8T cells by multiple immune cell infiltration algorithms (Fig. 6D). The results showed that SHMT2 was significantly negatively correlated with CD8T cell infiltration in several tumors, including HNSCC. We also analyzed the communication targets CD44, CXCR4 and CD74 in relation to MIF in HNSCC, and found that CD44 was stably negatively correlated with MIF in HNSCC (Fig. 6E). In conclusion, our results suggest that SHMT2 inhibits CD8T cell infiltration and induces immune escape by regulating the MIF/CD44 signaling axis, which in turn promotes HNSCC progression.

### Verification of the expression of SHMT2 in tissues and cell lines of HNSCC

**Fig. 7:**
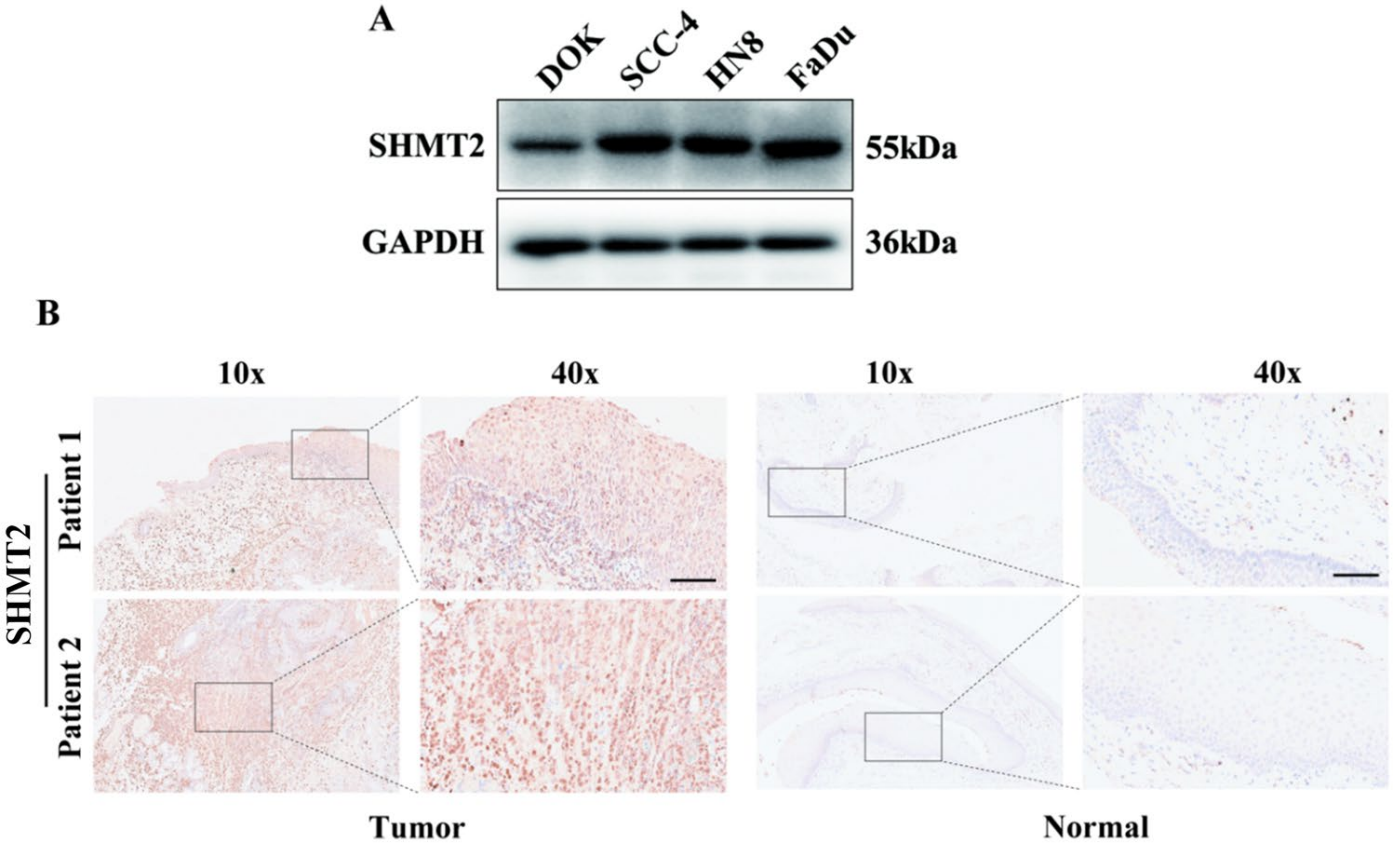
Verification of the expression of SHMT2 in tissues and cell lines of HNSCC. **A** Western blotting analysis of SHMT2 protein expression in DOK and three HNSCC cell lines (SCC-4, HN8 and FaDu). **B** Immunohistochemical analysis of SHMT2 protein expression in laryngocarcinoma and non-tumor tissues. The scale bar represents 100 μm

We further validated the above observation in our patient cohort using western blotting (Fig. 7A) and immunohistochemistry (Fig. 7B) of tumor and normal frozen tissues. The SHMT2 was obviously expressed in the three HNSCC cell lines (SCC-4, HN8 and FaDu) comparing to the normal DOK cell line. Similarly, compared to the normal tissues from larynx, the strong expression of SHMT2 can be observed in the laryngocarcinoma.

## Discussion

Folate metabolism plays an important role in DNA synthesis and methylation reactions by providing one-carbon units (Kim 2020; Zarou et al. 2021). Tumor cells use this mechanism to promote tumor growth through reprogramming of folate metabolism. Some studies have shown that disorders of folate metabolism are associated with the development and progression of several cancers(Kim 2020) and that high serum levels of folate portend a poor prognosis(Ben Fradj et al. 2021). However, some studies have also concluded that high intake of folic acid reduces the risk of developing BLCA (He and Shui 2014). This suggests that the mechanism of folate’s role in tumors needs to be further explored, especially in HNSCC. Our study found that reprogramming of folate metabolism in HNSCC suggested a poor prognosis, with higher folate levels being associated with a poorer prognosis, which is in keeping with previous findings. On the other hand, folate reduced pro-inflammatory cytokine secretion such as CCL2, CSF1, IL1A, IL6, IL10, and TNF-α. Immunosuppressive M2 macrophages led to reduced tumor immune surveillance and other tumor-infiltrating leukocytes interacted with cancer cells and activated the hypercomplex mechanism (Paijens et al. 2021; Samaniego et al. 2014; Sierra-Filardi et al. 2011). Folate metabolism has been studied more in immunity but less in HNSCC. Our study found a significant negative correlation between folate score and CD8T cell infiltration in HNSCC, with higher folate scores being associated with lower CD8T cell infiltration scores. Reprogramming of folate metabolism inhibits CD8T cell infiltration, which in turn promotes HNSCC progression. In summary, this study highlights the prognostic value of folate metabolic pathway, revealing some prognostic metabolic enzymes that can be used to predict the survival of HNSCC patients. Risk models based on these metabolic enzymes can serve as prognostic markers in clinical practice. These findings may provide new potential prognostic and therapeutic implications for the treatment of HNSCC patients.

The search for biomarkers that accurately and reliably predict prognosis and treatment outcome has been a hot topic. Identification of biomarkers from specific aspects of cell biology, such as autophagy, hypoxia, lipid metabolism and tumor immunity, has led to a deeper understanding of pathogenesis (Jiang et al. 2022; Wang et al. 2023; Zhou et al. 2023a). Although many predictive signatures have been constructed in tumors, no genetic signatures related to folate metabolism have been identified so far, especially in HNSCC. We developed a folate metabolism score consisting of six genes that objectively reflects the level of folate metabolism and accurately predicts patient prognosis. Xing et al. (Xing et al. 2020) developed a metabolism-based prognostic risk score for HNSCC using Cox regression analysis, which is not only a promising predictor of prognosis and survival, but also a potential biomarker for monitoring treatment plans. Our study was consistent with Liu’s findings and further emphasize the prognostic value of metabolic gene sets in HNSCC. MTHFD1L has been associated with colon cancer cell proliferation, colony formation, and invasion (Agarwal et al. 2019). MTHFD1L plays a major role in immune cell infiltration and prognostic biomarkers in patients with LIHC (Chen et al. 2021b). Increased expression of MTHFD1L has been associated with poor prognosis as well as DC, M0 macrophages and increased levels of immune infiltration in M2 macrophages. MTHFD2 contributes to tumorigenesis and immune evasion in a variety of cancers, and induces immune escape through upregulation of PD-L1 in pancreatic and bladder cancers (Li et al. 2023; Shang et al. 2021). In addition, MTHFD2/2L is a metabolic checkpoint that promotes Th17 metabolism by inhibiting mTORC1 in Treg cells (Sugiura et al. 2022). ATIC inhibits autophagy and promotes proliferation, invasion, and metastasis of hepatocellular carcinoma cells through the AKT/FOXO3 signaling pathway (Zhang et al. 2021).

Our study contributes to the development of novel biomarkers in HNSCC. SHMT2 is upregulated in several cancers and correlates with tumor progression (Chen et al. 2021a; DeNicola et al. 2015). For example, inhibition of SHMT2 in lymphomas induces alterations in DNA and histone methylation, which promotes lymphatic injury (Parsa et al. 2020). SHMT2 expression was positively correlated with advanced pathological grading of oral squamous cell carcinoma (Zhang et al. 2022). However, the function and specific mechanism of SHMT2 in HNSCC remain to be explored in depth. Consistent with our previous findings, we found that SHMT2 was significantly overexpressed in several tumors including HNSCC. More importantly, we validated this observation in our patient cohort using western blotting and immunohistochemistry of tumor and normal frozen tissues and similarly observed significant overexpression of SHMT2 in HNSCC. The higher SHMT2 expression suggests a worse prognosis for patients. The higher SHMT2 expression suggests that patients have a poorer prognosis, and SHMT2 was significantly and negatively correlated with CD8T cell infiltration in a variety of tumors, including HNSCC. We also found that CTL infiltration in the SHMT2 low-expression group suggested a good prognosis, whereas CTL infiltration in the SHMT2 high-expression group suggested a poor prognosis, suggesting that SHMT2 is associated with T cell depletion. Several studies have reported that SHMT2 is significantly and positively correlated with PD-L1 expression (Wu et al. 2019; Zhou et al. 2023b). PD-L1 inhibits T cell responses by activating the downstream signaling pathway of the PD-1 receptor, reducing T cell activity and promoting apoptosis, thereby inhibiting T cell responses and leading to tumor immune escape. This is in keeping with our findings.

As a multifunctional cytokine, MIF mainly interacts with membrane receptors CXC family, CD74 and CD44 to activate the downstream signaling pathway and exert biological functions. The membrane protein CD74 is the high-affinity receptor of MIF, and MIF can regulate the activity of CD74, causing homeostatic disorders such as inflammation, tumor and autoimmune diseases (Pantouris et al. 2018). MIF binds to the chemokines CXCR2 and CXCR4, recruits leukocytes, and accelerates the process of atherosclerosis (Rajasekaran et al. 2016). Further studies revealed that these membrane receptors usually bind to MIF in the form of complexes (e.g., CXCR4/CD74, CXCR2/CD74, and CD74/CD44) to regulate cellular functions, e.g., MIF binds to CD74/CD44 complexes to activate signals such as Syk, Akt, and NF-κB, and regulate immune responses (Gore et al. 2008). Our study found that there was significant cellular communication between tumor cells and CD8T cells in HNSCC, and the MIF signaling pathway was significantly enriched. We found that MIF was significantly overexpressed in several tumors, including HNSCC, and the higher MIF expression suggested that patients had a worse prognosis. The higher the expression of MIF, the worse the prognosis of patients, and MIF was positively correlated with SHMT2 and negatively correlated with CD44 and CD8 T cell infiltration in many tumors, including HNSCC. Recent studies have shown that MIF-CD74 can inhibit anti-tumor immune responses by recruiting tumor-associated macrophages (TAMs) or directly inhibiting T cell activation (Balogh et al. 2018; Klemke et al. 2021). CD44 plays an important role in T cell activation and memory responses, and CD44 interacts with other surface receptors (e.g. TCRs and co-stimulatory molecules) to enhance activation signaling of T cells and promote cell proliferation and function fulfillment. Our study found that in HNSCC, SHMT2 suppresses anti-tumor immune responses by down-regulating CD44 expression and inhibiting T cell activation through MIF.

Multiple methods, databases, and bioinformatics analyses were utilized to explore the potential function of folate metabolism in HNSCC. SHMT2 may be a potential prognostic marker for improving survival and prognostic accuracy in HNSCC patients, and may even be a potential biomarker for HNSCC patients, indicating response to immunotherapy. However, this study was based on an open database; therefore, further studies are needed to investigate the mechanism of action of SHMT2 in HNSCC.

## Conclusion

Our study identified a novel and robust folate metabolic signature. A folate metabolic signature comprising six genes was effective in assessing the prognosis and reflecting the immune status of HNSCC patients. The folate metabolic signature may be involved in the regulation of immune-related signaling pathways, providing a promising target for improving prognosis and HNSCC response to immunotherapy. Our results showed that SHMT2, an independent prognostic marker, was highly expressed in HNSCC patients. In addition, SHMT2 is involved in certain processes of tumorigenesis and development and is associated with the expression of MIF, CD74, CXCR4 and CD44 in the HNSCC tumor microenvironment. More studies are needed on the role of SHMT2 as an enzyme involved in folate metabolism in the HNSCC microenvironment and the specific mechanisms between SHMT2 and the HNSCC immune microenvironment. Future potential research directions aim to: investigate the specific mechanisms of SHMT2 in immune-related signaling pathways in HNSCC, and its impact on therapeutic immune responses; and evaluate the clinical significance of targeting SHMT2 in HNSCC treatment and its role as a therapeutic intervention to improve patient prognosis.

## Supplementary Information

Below is the link to the electronic supplementary material.

Supplementary file1 (DOCX 168 KB)

Supplementary file2 (XLSX 67 KB)

Supplementary file3 (XLSX 10 KB)

## Data Availability

The dataset was downloaded from the TCGA database (https://tcga-data.nci.nih.gov/tcga/), and GSE103322 were downloaded from the GEO database (http://www.ncbi.nlm.nih.gov/geo/).

## References

[CR1] Agarwal S, Behring M, Hale K, Al Diffalha S, Wang K, Manne U (2019) MTHFD1L, A folate cycle enzyme, is involved in progression of colorectal cancer. Transl Oncol 12(11):1461–1467 31421459 10.1016/j.tranon.2019.07.011 PMC6704443

[CR2] Anderson DD, Quintero CM, Stover PJ (2011) Identification of a de novo thymidylate biosynthesis pathway in mammalian mitochondria. Proc Natl Acad Sci U S A 108(37):15163–15168 21876188 10.1073/pnas.1103623108 PMC3174652

[CR3] Balogh KN, Templeton DJ, Cross JV (2018) Macrophage Migration Inhibitory Factor protects cancer cells from immunogenic cell death and impairs anti-tumor immune responses. PLoS ONE 13(6):e0197702 29864117 10.1371/journal.pone.0197702 PMC5986154

[CR4] Ben Fradj MK, Ouanes Y, Hadj-Taeib S, Mrad Dali K, Bibi M, Jmal K (2021) Prognostic significance of plasma folate and cobalamin concentrations in non-muscle-invasive bladder cancer: a prospective cohort study. Cancer Invest 39(3):240–250 33074725 10.1080/07357907.2020.1839760

[CR5] Chen J, Na R, Xiao C, Wang X, Wang Y, Yan D (2021a) The loss of SHMT2 mediates 5-fluorouracil chemoresistance in colorectal cancer by upregulating autophagy. Oncogene 40(23):3974–3988 33990700 10.1038/s41388-021-01815-4 PMC8195740

[CR6] Chen J, Yang J, Xu Q, Wang Z, Wu J, Pan L (2021) Integrated bioinformatics analysis identified MTHFD1L as a potential biomarker and correlated with immune infiltrates in hepatocellular carcinoma. Biosci Rep. 10.1042/BSR20202063 10.1042/BSR20202063 PMC7897918 33605411

[CR7] Cohen EE, LaMonte SJ, Erb NL, Beckman KL, Sadeghi N, Hutcheson KA (2016) American cancer society head and neck cancer survivorship care guideline. CA Cancer J Clin 66(3):203–239 27002678 10.3322/caac.21343

[CR8] Cole BF, Baron JA, Sandler RS, Haile RW, Ahnen DJ, Bresalier RS (2007) Folic acid for the prevention of colorectal adenomas: a randomized clinical trial. JAMA 297(21):2351–2359 17551129 10.1001/jama.297.21.2351

[CR9] DeNicola GM, Chen PH, Mullarky E, Sudderth JA, Hu Z, Wu D (2015) NRF2 regulates serine biosynthesis in non-small cell lung cancer. Nat Genet 47(12):1475–1481 26482881 10.1038/ng.3421 PMC4721512

[CR10] Ding J, Li T, Wang X, Zhao E, Choi JH, Yang L (2013) The histone H3 methyltransferase G9A epigenetically activates the serine-glycine synthesis pathway to sustain cancer cell survival and proliferation. Cell Metab 18(6):896–907 24315373 10.1016/j.cmet.2013.11.004 PMC3878056

[CR11] Ducker GS, Rabinowitz JD (2017) One-carbon metabolism in health and disease. Cell Metab 25(1):27–42 27641100 10.1016/j.cmet.2016.08.009 PMC5353360

[CR12] Ebbing M, Bonaa KH, Nygard O, Arnesen E, Ueland PM, Nordrehaug JE (2009) Cancer incidence and mortality after treatment with folic acid and vitamin B12. JAMA 302(19):2119–2126 19920236 10.1001/jama.2009.1622

[CR13] Ferlay J, Soerjomataram I, Dikshit R, Eser S, Mathers C, Rebelo M (2015) Cancer incidence and mortality worldwide: sources, methods and major patterns in GLOBOCAN 2012. Int J Cancer 136(5):E359–E386 25220842 10.1002/ijc.29210

[CR14] Figueiredo JC, Grau MV, Haile RW, Sandler RS, Summers RW, Bresalier RS (2009) Folic acid and risk of prostate cancer: results from a randomized clinical trial. J Natl Cancer Inst 101(6):432–435 19276452 10.1093/jnci/djp019 PMC2657096

[CR15] Gore Y, Starlets D, Maharshak N, Becker-Herman S, Kaneyuki U, Leng L (2008) Macrophage migration inhibitory factor induces B cell survival by activation of a CD74-CD44 receptor complex. J Biol Chem 283(5):2784–2792 18056708 10.1074/jbc.M703265200

[CR16] He H, Shui B (2014) Folate intake and risk of bladder cancer: a meta-analysis of epidemiological studies. Int J Food Sci Nutr 65(3):286–292 24328495 10.3109/09637486.2013.866641

[CR17] Jiang L, Chen S, Pan Q, Zheng J, He J, Sun J (2022) The feasibility of proteomics sequencing based immune-related prognostic signature for predicting clinical outcomes of bladder cancer patients. BMC Cancer 22(1):676 35725413 10.1186/s12885-022-09783-y PMC9210750

[CR18] Jin Y, Jung SN, Lim MA, Oh C, Piao Y, Kim HJ (2022) SHMT2 induces stemness and progression of head and neck cancer. Int J Mol Sci 23(17):9714 36077112 10.3390/ijms23179714 PMC9456418

[CR19] Johnson DE, Burtness B, Leemans CR, Lui VWY, Bauman JE, Grandis JR (2020) Head and neck squamous cell carcinoma. Nat Rev Dis Primers 6(1):92 33243986 10.1038/s41572-020-00224-3 PMC7944998

[CR20] Kim YI (2007) Folate and colorectal cancer: an evidence-based critical review. Mol Nutr Food Res 51(3):267–292 17295418 10.1002/mnfr.200600191

[CR21] Kim SE (2020) Enzymes involved in folate metabolism and its implication for cancer treatment. Nutr Res Pract 14(2):95–101 32256983 10.4162/nrp.2020.14.2.95 PMC7075736

[CR22] Klemke L, De Oliveira T, Witt D, Winkler N, Bohnenberger H, Bucala R (2021) Hsp90-stabilized MIF supports tumor progression via macrophage recruitment and angiogenesis in colorectal cancer. Cell Death Dis 12(2):155 33542244 10.1038/s41419-021-03426-z PMC7862487

[CR23] Lee GY, Haverty PM, Li L, Kljavin NM, Bourgon R, Lee J (2014) Comparative oncogenomics identifies PSMB4 and SHMT2 as potential cancer driver genes. Cancer Res 74(11):3114–3126 24755469 10.1158/0008-5472.CAN-13-2683

[CR24] Li L, Zhang Y, Hu W, Zou F, Ning J, Rao T (2023) MTHFD2 promotes PD-L1 expression via activation of the JAK/STAT signalling pathway in bladder cancer. J Cell Mol Med 27(19):2922–2936 37480214 10.1111/jcmm.17863 PMC10538262

[CR25] Paijens ST, Vledder A, de Bruyn M, Nijman HW (2021) Tumor-infiltrating lymphocytes in the immunotherapy era. Cell Mol Immunol 18(4):842–859 33139907 10.1038/s41423-020-00565-9 PMC8115290

[CR26] Pantouris G, Ho J, Shah D, Syed MA, Leng L, Bhandari V (2018) Nanosecond dynamics regulate the MIF-induced activity of CD74. Angew Chem Int Ed Engl 57(24):7116–7119 29669180 10.1002/anie.201803191 PMC6282165

[CR27] Parsa S, Ortega-Molina A, Ying HY, Jiang M, Teater M, Wang J (2020) The serine hydroxymethyltransferase-2 (SHMT2) initiates lymphoma development through epigenetic tumor suppressor silencing. Nat Cancer 1:653–664 33569544 10.1038/s43018-020-0080-0 PMC7872152

[CR28] Rajasekaran D, Groning S, Schmitz C, Zierow S, Drucker N, Bakou M (2016) Macrophage migration inhibitory factor-CXCR4 receptor interactions: evidence for partial allosteric agonism in comparison with CXCL12 CHEMOKINE. J Biol Chem 291(30):15881–15895 27226569 10.1074/jbc.M116.717751 PMC4957068

[CR29] Samaniego R, Palacios BS, Domiguez-Soto A, Vidal C, Salas A, Matsuyama T (2014) Macrophage uptake and accumulation of folates are polarization-dependent in vitro and in vivo and are regulated by activin A. J Leukoc Biol 95(5):797–808 24399840 10.1189/jlb.0613345

[CR30] Shang M, Yang H, Yang R, Chen T, Fu Y, Li Y (2021) The folate cycle enzyme MTHFD2 induces cancer immune evasion through PD-L1 up-regulation. Nat Commun 12(1):1940 33782411 10.1038/s41467-021-22173-5 PMC8007798

[CR31] Sierra-Filardi E, Puig-Kroger A, Blanco FJ, Nieto C, Bragado R, Palomero MI (2011) Activin A skews macrophage polarization by promoting a proinflammatory phenotype and inhibiting the acquisition of anti-inflammatory macrophage markers. Blood 117(19):5092–5101 21389328 10.1182/blood-2010-09-306993

[CR32] Stolzenberg-Solomon RZ, Chang SC, Leitzmann MF, Johnson KA, Johnson C, Buys SS (2006) Folate intake, alcohol use, and postmenopausal breast cancer risk in the Prostate, Lung, Colorectal, and Ovarian Cancer Screening Trial. Am J Clin Nutr 83(4):895–904 16600944 10.1093/ajcn/83.4.895

[CR33] Sugiura A, Andrejeva G, Voss K, Heintzman DR, Xu X, Madden MZ (2022) MTHFD2 is a metabolic checkpoint controlling effector and regulatory T cell fate and function. Immunity 55(1):65-81 e9 34767747 10.1016/j.immuni.2021.10.011 PMC8755618

[CR34] Tibbetts AS, Appling DR (2010) Compartmentalization of Mammalian folate-mediated one-carbon metabolism. Annu Rev Nutr 30:57–81 20645850 10.1146/annurev.nutr.012809.104810

[CR35] Wang H, Yang C, Li D, Wang R, Li Y, Lv L (2023) Bioinformatics analysis and experimental validation of a novel autophagy-related signature relevant to immune infiltration for recurrence prediction after curative hepatectomy. Aging (Albany NY) 15(7):2610–2630 37014321 10.18632/aging.204632 PMC10120910

[CR36] Wu Y, Chen W, Xu ZP, Gu W (2019) PD-L1 distribution and perspective for cancer immunotherapy-blockade, knockdown, or inhibition. Front Immunol 10:2022 31507611 10.3389/fimmu.2019.02022 PMC6718566

[CR37] Xing L, Guo M, Zhang X, Zhang X, Liu F (2020) A transcriptional metabolic gene-set based prognostic signature is associated with clinical and mutational features in head and neck squamous cell carcinoma. J Cancer Res Clin Oncol 146(3):621–630 32067104 10.1007/s00432-020-03155-4 PMC11804320

[CR38] Zarou MM, Vazquez A, Vignir HG (2021) Folate metabolism: a re-emerging therapeutic target in haematological cancers. Leukemia 35(6):1539–1551 33707653 10.1038/s41375-021-01189-2 PMC8179844

[CR39] Zhang H, Xia P, Liu J, Chen Z, Ma W, Yuan Y (2021) ATIC inhibits autophagy in hepatocellular cancer through the AKT/FOXO3 pathway and serves as a prognostic signature for modeling patient survival. Int J Biol Sci 17(15):4442–4458 34803509 10.7150/ijbs.65669 PMC8579461

[CR40] Zhang H, Che Y, Xuan B, Wu X, Li H (2022) Serine hydroxymethyltransferase 2 (SHMT2) potentiates the aggressive process of oral squamous cell carcinoma by binding to interleukin enhancer-binding factor 2 (ILF2). Bioengineered 13(4):8785–8797 35333683 10.1080/21655979.2022.2051886 PMC9161932

[CR41] Zhou R, Liang J, Chen Q, Tian H, Yang C, Liu C (2023a) A 3-gene random forest model to diagnose non-obstructive azoospermia based on transcription factor-related henes. Reprod Sci 30(1):233–246 35715550 10.1007/s43032-022-01008-8

[CR42] Zhou YJ, Li G, Wang J, Liu M, Wang Z, Song Y (2023b) PD-L1: expression regulation. Blood Sci 5(2):77–91 37228770 10.1097/BS9.0000000000000149 PMC10205351

