# Supplementary material for: Construction and validation of a folate metabolism-related gene signature for predicting prognosis in HNSCC
Source: J Cancer Res Clin Oncol. 2024 Apr 16;150(4):198. doi: 10.1007/s00432-024-05731-4 (PMC11021263; doi:10.1007/s00432-024-05731-4)
Supplement: Supplementary file 1 — Supplementary file1 (DOCX 168 KB) [file 432_2024_5731_MOESM1_ESM.docx]

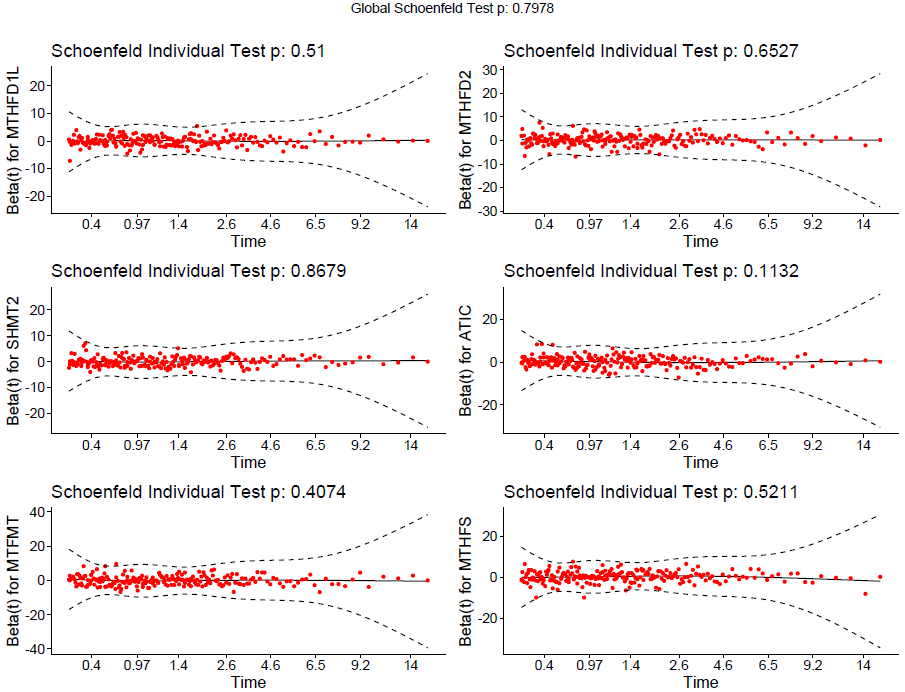


**Supplementary Figure 1**. Schoenfeld residuals for assessing the proportional hazards assumption in the Cox regression models.


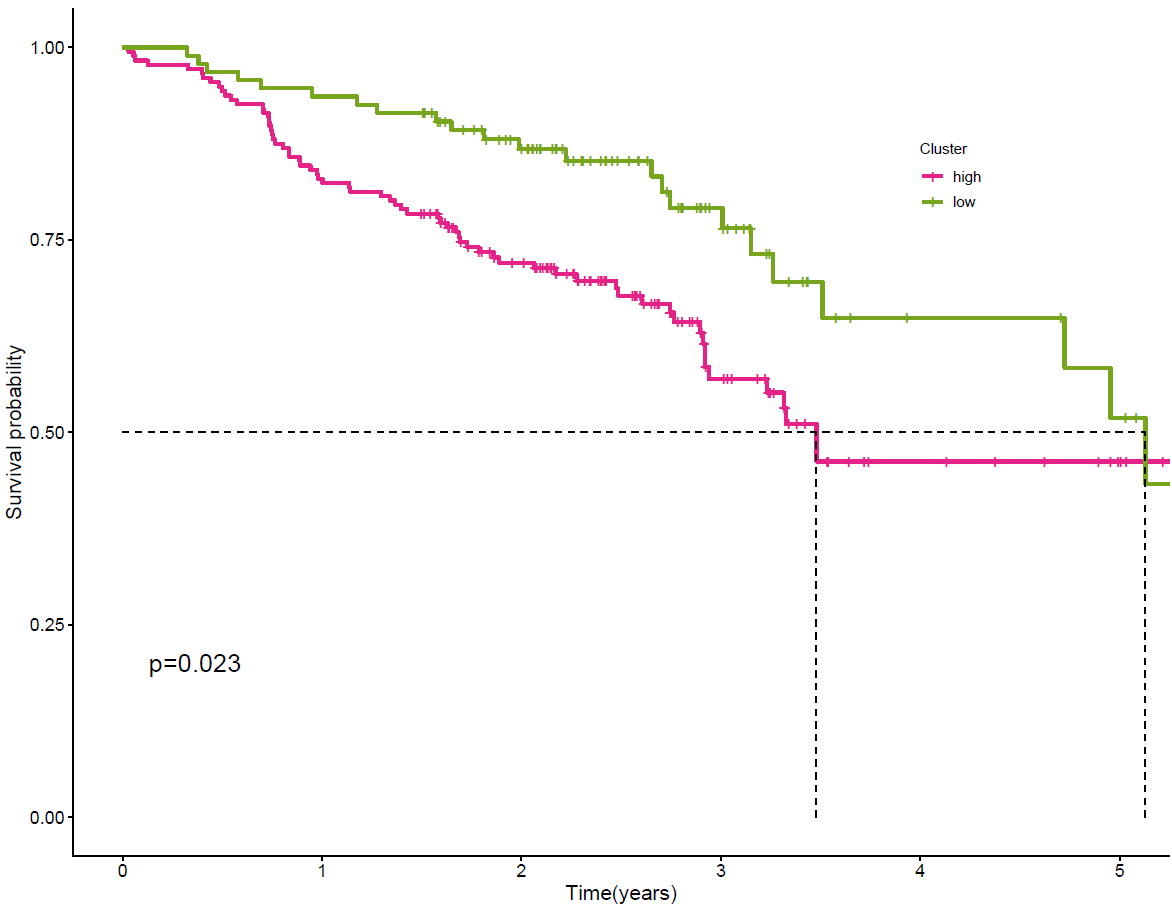


**Supplementary Figure 2**. K-M curves for folate metabolism scores in GSE65858.
